# Mucins in pancreatic cancer: A well‐established but promising family for diagnosis, prognosis and therapy

**DOI:** 10.1111/jcmm.15684

**Published:** 2020-08-03

**Authors:** Shunda Wang, Lei You, Menghua Dai, Yupei Zhao

**Affiliations:** ^1^ Department of General Surgery Peking Union Medical College Hospital Chinese Academy of Medical Sciences & Peking Union Medical College Beijing China

**Keywords:** diagnosis, mucins, pancreatic cancer, prognosis, therapy

## Abstract

Mucins are a family of multifunctional glycoproteins that mostly line the surface of epithelial cells in the gastrointestinal tract and exert pivotal roles in gut lubrication and protection. Pancreatic cancer is a lethal disease with poor early diagnosis, limited therapeutic effects, and high numbers of cancer‐related deaths. In this review, we introduce the expression profiles of mucins in the normal pancreas, pancreatic precursor neoplasia and pancreatic cancer. Mucins in the pancreas contribute to biological processes such as the protection, lubrication and moisturization of epithelial tissues. They also participate in the carcinogenesis of pancreatic cancer and are used as diagnostic biomarkers and therapeutic targets. Herein, we discuss the important roles of mucins that lead to the lethality of pancreatic adenocarcinoma, particularly MUC1, MUC4, MUC5AC and MUC16 in disease progression, and present a comprehensive analysis of the clinical application of mucins and their promising roles in cancer treatment to gain a better understanding of the role of mucins in pancreatic cancer.

## 1 INTRODUCTION

Pancreatic cancer (PC) is the fourth leading cause of cancer‐related deaths in the United States and has a 5‐year survival rate of <6%.^1^ Pancreatic ductal adenocarcinoma (PDAC) remains the most common type, and nearly 250 000 new cases are diagnosed per year worldwide.^2^ In China, it ranks sixth among all cancer types.^3^ It is reported that the mortality rate of PC will become the second highest among cancer‐related deaths before 2030.^4^ PDAC is not yet fully understood by clinicians and scientists, and its major symptoms, including abdominal pain, obstructive jaundice and Cullen syndrome, are not easily detected at the early stages. Surgery is the only effective treatment for PDAC patients, but most patients miss the optimal time of surgery because of the late diagnosis.^5^ Chemotherapy and radiotherapy may improve the poor prognosis slightly but with severe side effects.^3^ Despite the increasing research efforts by cancer scientists, there is an urgent need for breakthroughs on the mechanism of oncogenesis and targeted therapy.

**FIGURE 1:**
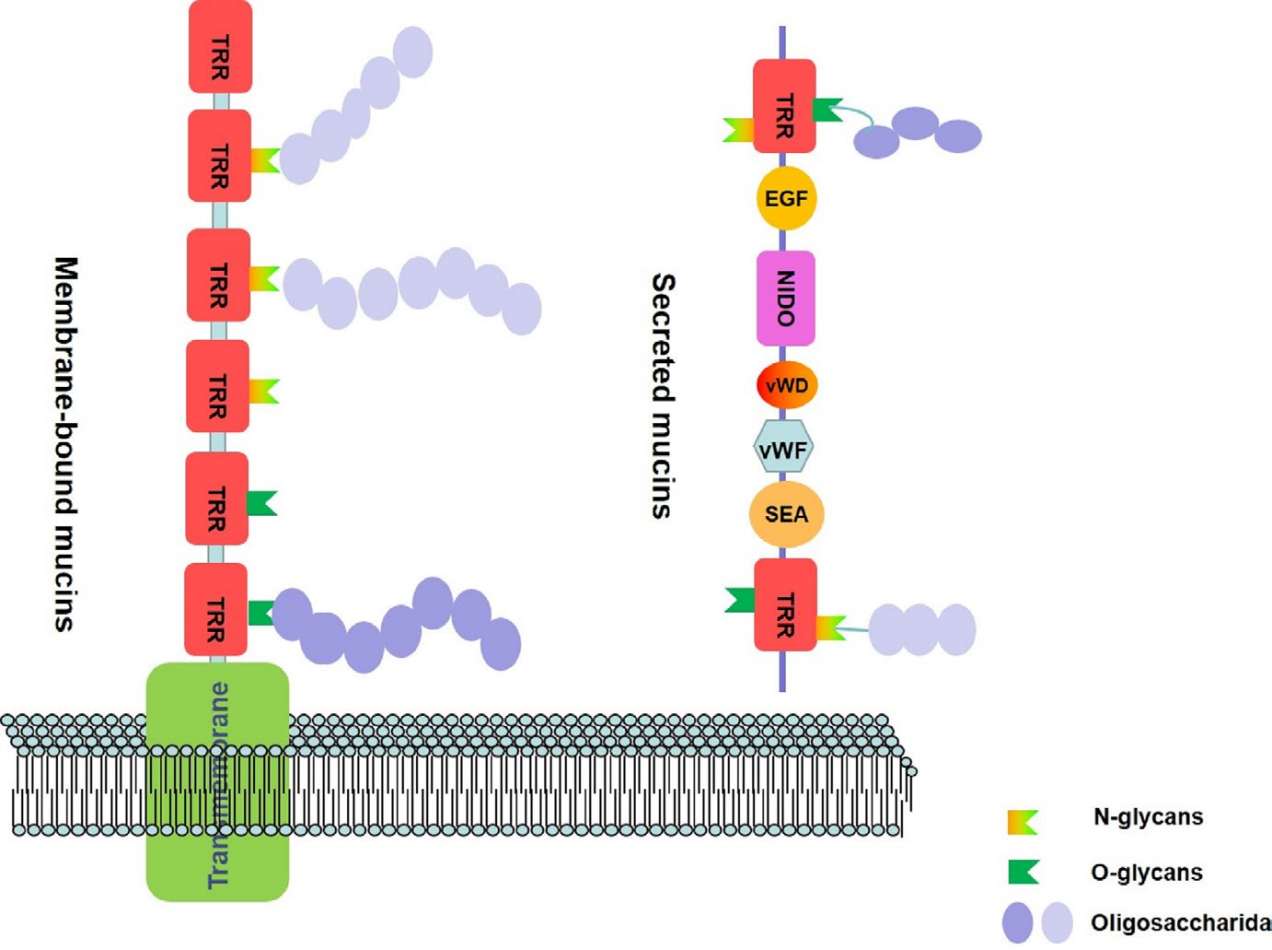
The structure of membrane‐bound mucin and secreted mucin. EGF, epidermal growth factor; NIDO, nidogen‐like domain; SEA, sea urchin sperm protein‐enterokinase‐agrin; TRR, tandem repeat region; vWD, von Willebrand factor D domain; vWF, von Willebrand factor like domain

Early diagnosis is clinically dependent on one biomarker, carbohydrate antigen 19‐9 (CA19‐9). CA19‐9, an epitope of sialylated Lewis group antigen, is elevated in approximately 80% of patients. Patients diagnosed with locally advanced or metastatic disease usually have much higher levels of CA19‐9.^2^ Antigens such as CA19‐9 are composed of oligosaccharide structures present on heavily glycosylated mucins born by the antigen.^6^ Mucins are produced by various epithelial cells that are located on serine or threonine residues of the mucin core protein backbone. There are repetitive short stretches in the protein backbone termed tandem repeat regions (TRRs), while many O‐glycosidic or N‐glycosidic linkages are connected to the backbone.^7^ Furthermore, there are multiple functional domains in mucin family members including von Willebrand factor D domain (vWD), nidogen‐like domain (NIDO), von Willebrand factor like domain (vWF‐like), epidermal growth factor (EGF) and sea urchin sperm protein‐enterokinase‐agrin (SEA). These extended structures are used for communication with cellular receptors, extracellular matrix and signalling mediators^8^, ^9^ (Figure 1).

Genes encoding mucins are denoted by the first three letters, “MUC,” followed by the number in which they were discovered chronologically.^10^ Mucins are divided into two categories: membrane‐bound mucins and secreted mucins. The first class includes MUC1, MUC3, MUC4 and MUC16, which have been frequently investigated. MUC12, MUC13, MUC17 and MUC21 are also included in this group but limited studies have been performed on these. The protein structure of these mucins is composed of an N‐terminal portion and a transmembrane domain, which has many phosphorylation sites that are involved in signal transduction.^10^, ^11^ The latter group of secreted mucins include MUC2, MUC5AC, MUC5B, MUC6 and MUC7, which are important for the formation of mucin heterodimers or homodimers.^9^ Furthermore, another mucin, MUC18 (melanoma cell adhesion molecule), belongs to the immunoglobulin superfamily.^10^ As well as protecting the barriers of mucous membranes, mucins also have important roles in cellular regeneration, differentiation, migration, adhesion and signalling.^8^, ^12^

Mucins may represent a diagnostic parameter for the early detection of PC and act as specific discriminated biomarkers among PC, pancreatic intraepithelial neoplasia (PanIN) and pancreatitis. Our review aims to summarize and update the role of mucins in the pathogenesis of PC and focuses on the diagnostic, prognostic and therapeutic functions of these glycoproteins in PDAC.

## 2 MUCINS IN THE NORMAL PANCREAS

Exploring the distribution of certain mucins in normal pancreatic tissue may help to determine their biological significance. Much research has focused on mucin expression in normal and pathological pancreatic tissue to reveal a significant role in cancer pathogenesis.^11^, ^12^, ^13^, ^14^ MUC1 has been widely studied for many years because of its oncogenic features in various cancers including PC.^15^ In normal pancreatic tissue, MUC1 is expressed in the intralobular ducts and centrally in the interlobular ducts, while the glycoforms of MUC1 are undetectable in the main pancreatic duct. MUC2 and MUC4 expression is also undetectable in the normal pancreas.^16^ MUC3 expressed in the main pancreatic duct is reduced to low levels at 13 weeks of gestation, and MUC6 can be detected from 13 weeks in the pancreatic duct system, and is mainly detected in small interlobular ducts and developing acini,^7^ and also in normal adult pancreas tissue.^16^, ^17^, ^18^ Furthermore, while MUC5AC is not detectable, MUC5B is found in normal pancreatic tissue and the normal pancreatic duct,^5^, ^13^, ^14^ unlike MUC7, which is also undetectable in the normal pancreas.^7^ Other mucin genes, such as MUC11, MUC12 and MUC17 can be detected in normal pancreatic tissues. However, their specific expression pattern in the development of PC is unclear.^19^, ^20^ It was reported that there is relatively low expression of MUC20 and MUC21 in normal pancreatic tissue.^21^, ^22^

## 3 MUCINS IN PRECURSOR LESIONS OF THE PANCREAS

Pancreatic ductal adenocarcinoma is the major type of pancreatic malignancy and the transformation model for this disease recently focused on the concept of intraepithelial neoplasia, termed PanIN. According to the morphological pattern, PanINs are divided into PanIN‐1, PanIN‐2 and PanIN‐3 based on the different cellular morphologies and architectural atypia.^23^ The profile of mucin expression in the pancreas is affected by the pathological conditions, and mucin exerts individualized functions in the development of premalignant and malignant neoplasms. MUC1 is reported to be expressed in the apical membrane of the intralobular duct in normal pancreatic tissue. However, aberrant MUC1 expression can be detected during the early stages of PC and gradually increases with the formation of invasive carcinoma.^11^, ^24^, ^25^ Other studies revealed that *MUC1* RNA levels are low in the tissue of both chronic pancreatitis and normal pancreas, while high MUC1 expression indicates the development of PC or PanIN progression.^5^ As for mucin expression in intraductal papillary‐mucinous neoplasm (IPMN), MUC1 tends to promote the development of PDAC and the potential for metastasis.^26^ Similarly, MUC4 is minimally expressed in PanIN and chronic pancreatitis but is highly expressed in PC cell lines,^14^, ^27^ and aberrant overexpression of MUC4 was also associated with the progression of PanIN to PC,^28^, ^29^ while the intensity of expression increased in parallel with the severity of cellular dysplasia.^29^ MUC5B is weakly expressed in pancreatic precursor lesions, while MUC5AC is gradually expressed during PanIN transformation. Since MUC5AC is seldomly expressed in the normal pancreas,^30^ it has value as a biomarker of early neoplastic lesions, which correlate with poor prognosis.^31^ Although MUC6 can be detected in the foetal pancreas in the early period of gestation,^32^, ^33^ it was reported have no involvement in the prognosis of PC patients.^34^

## 4 MUCINS IN PANCREATIC CANCER

Mucins form a protective coat around cancer cells and have critical roles in the carcinogenesis of PC.^35^ Several studies of the abnormal expression of mucins in PDAC indicated their potential roles in its pathogenesis, such as restricting intracellular drug uptake, facilitating immune escape, up‐regulating signal pathways or even serving as prognostic biomarkers.^36^, ^37^, ^38^ PDAC is characterized by the differential expression or glycosylation of the mucin family as it transforms from healthy tissue to cancer, as outlined in Table 1.

**TABLE 1:** Mucins expression in different pancreatic neoplasms

	Normal	PanIN			Pancreatic cancer	Pancreatitis	IPMN	MCN
		I	II	III				
MUC1	+	+	++	+++	+++	++	±	±
MUC2	−	−	−	−	−	−	++	NA
MUC3	±	+	+	+	++	±	±	NA
MUC4	−	+	++	+++	+++	−	±	++
MUC5AC	−	+	++	+++	+++	±	+	+
MUC5B	±	NA	NA	NA	+++	++	±	NA
MUC6	+	++	++	++	+++	++	++	NA
MUC7	NA	NA	NA	NA	+	+	NA	NA
MUC11/12	±	NA	NA	NA	NA	NA	NA	NA
MUC13	±	NA	NA	NA	+++	NA	NA	NA
MUC16	−	+	++	++	+++	NA	NA	NA
MUC17	+	++	++	++	+	NA	NA	NA
MUC20	+	NA	NA	NA	NA	NA	NA	NA
MUC21	+	NA	NA	NA	NA	NA	NA	NA

Abbreviations: −, negative; +, low; ++, moderate; +++, intense; ±, negative or positive depending the reports; IPMN, intraductal papillary‐mucinous neoplasms; MCN, mucinous cystic neoplasm; NA, no available data; PanIN, pancreatic intra‐epithelial neoplasia.

### 4.1 MUC1

Many studies have focused on *MUC1* as an oncogene, not only in PDAC but also in different cancer types.^39^, ^40^, ^41^ MUC1 is expressed mainly along on the luminal cell surface and partially along the baso‐lateral surface, and is detected in PDAC exhibiting aggressive behaviour.^42^ High MUC1 expression is detected in more than 60% of PC cases and is significantly associated with tumour size. Furthermore, the higher the level of MUC1 expression, the poorer the prognosis in patients with PC.^43^ In addition, PC manifests high expression rates of MUC1 glycoforms (MUC1/MY.1E12, 98%; MUC1/DF3, 96%; MUC1/HMFG1, 76%; and MUC1/CORE, 66%).^44^

The cytoplasmic tail of MUC1 (MUC1‐C) is widely involved in carcinogenesis and is correlated with PC progression by promoting the aggressive and metastatic phenotype.^45^ MUC1‐C contains multiple phosphorylated residues and interacts with signal transducers, cytokines, growth factors and other receptor tyrosine kinases to activate downstream signalling cascades.^46^ For example, integrin‐linked kinase, platelet‐derived growth factor receptor‐β and Met receptor tyrosine kinase modulate MUC1‐C and enhance its oncogenic properties in PC.^47^, ^48^, ^49^ Moreover, MUC1 in PC binds to β‐catenin and EGFR to activate cell proliferation via the Wnt/ß‐catenin or MAPK pathways.^50^, ^51^ MUC1 in PDAC significantly prompts tube formation, vessel generation and metastasis through the NRP1–VEGF axis^52^ and can also induce epithelial‐mesenchymal transition to facilitate metastasis.^51^

Additionally, because of the loss of apical‐basal polarity in cancers, aberrantly glycosylated MUC1 was demonstrated to regulate metabolism via several aspects, including directly influencing metabolic gene expression, modulating the activity of metabolic enzymes and even indirectly regulating reactive oxygen species (ROS) and autophagy.^53^, ^54^ The observations of MUC1‐induced metabolic alteration of PC have also strengthened its role as an oncoprotein. MUC1 interacting with HIF‐1α induced metabolic reprogramming to impart gemcitabine chemoresistance in PC.^54^, ^55^ Furthermore, MUC1 was reported to promote chemoresistance in PC cells by inhibiting BRCA1 to enhance glycolysis.^25^ MUC1 also has a role in response to radiotherapy by enhancing biosynthesis and glycolysis in PC cells to destroy radiation‐induced cytotoxicity.^56^ Moreover, glucose limitation in MUC1‐up‐regulated PC cells disrupts the synthesis of pyrimidine nucleotides and increases the consumption of glutamine, resulting in a metabolic switch.^57^ MUC1 oncoprotein could also facilitate the pro‐adaptive stress response axis through cytidine deaminase‐mediated pyrimidine metabolic reprogramming and ROS alterations, therefore regulating cancer cell survival and chemotherapy response in PC.^58^

### 4.2 MUC4

MUC4 is overexpressed in most PCs, or even as early as the PanIN‐I stages.^29^, ^59^ Several studies reported that MUC4 imparts several oncogenic properties and is detected in 32%–89% of PDAC patients.^28^, ^29^, ^60^ These differences can be attributed to the use of different MUC4‐positive thresholds. MUC4, regarded as the tumour‐associated mucin of the pancreas, might be a promising biomarker to discriminate PC from pancreatitis. The survival rate of PC patients with high MUC4 expression was significantly lower than that of patients with low MUC4 expression.^61^ Moreover, MUC4 acts as an important factor in modulating the chemoresistance of gemcitabine in PC cells. Down‐regulation of MUC4 can sensitize highly metastatic PC cells to gemcitabine in vitro.^62^ In addition, MUC4 was also found to be expressed in PC stem cells, indicating its important role not only in maintaining cell proliferation but also in increasing the chemoresistance of PC cells.^63^ Interestingly, MUC4 inhibits apoptosis by interfering with caspase protein and cytochrome C present in the mitochondria,^64^, ^65^ and MUC4/β‐catenin can suppress the progression and metastasis of PC by interfering GCNT3, a glycosyltransferase.^66^ Recently, it was identified as a new tumour‐related antigen for immunotherapy in PC. The recombinant MUC4 domain and the elicited cellular and humoral anti‐MUC4 response indicate its application as a candidate vaccine for PC therapy.^67^

### 4.3 MUC5AC

MUC5AC is highly expressed in different grades of PanIN and in IPMN and PDAC.^68^, ^69^ Among 132 cases of PDAC, 92% were positive for MUC5AC,^60^ *MUC5AC* mRNA expression was also higher in tumoral tissues compared with para‐tumoral tissue,^70^ which might be correlated with PDAC invasion, suggesting its role in the acceleration of cancer progression. In vivo xenograft research indicates that low levels of MUC5AC expression suppress tumorigenicity and inhibit neutrophil‐induced apoptosis.^71^ Down‐regulation of MUC5AC could also result in decreased growth and metastasis, while up‐regulation of MUC5AC accelerated high‐grade PanIN to invasive cancer.^72^ Furthermore, MUC5AC seems to be a sensitive biomarker of early pancreatic neoplasms, providing another link with unfavourable prognosis.^30^, ^31^ In addition, the combination of MUC5AC and CA19‐9 presented optimal performance and improved specificity compared with CA19‐9 to differentiate PC from healthy controls.^73^, ^74^ However, contrary results revealed that the expression level of MUC5AC is correlated with promising clinical events in PC.^75^, ^76^

### 4.4 MUC16

MUC16, also known as CA‐125 because it carries a CA‐125 epitope,^77^ is a widely expressed tumour antigen observed in ovarian cancer that is usually elevated in PC, although it has relatively low specificity. MUC16 was proven to exert a critical role in the formation, progression, metastases and relapse of PC.^78^, ^79^ MUC16 expression was found to be remarkably lower in low‐grade dysplasia compared with high‐grade dysplasia,^78^ while it was much higher in metastatic foci than that at primary sites; thus, we can conclude that it may have a pivotal function in the metastasis of PC.^79^ Clinically, it is also combined with CA19‐9 to evaluate the severity and prognosis of PC.^80^, ^81^ MUC16 is involved in many oncogenic signalling pathways, such as activating LMO2 and JAK2 to promote proliferation, and up‐regulation of MUC16 stimulates mTOR and c‐MYC to reprogram PC metabolism, enhancing glycolysis and cell proliferation.^82^, ^83^ MUC16 can also activate the AKT and MAPK pathways to promote metastasis,^83^ and the MUC16‐C terminal can promote the enrichment of Tregs through IL‐6 activation of the JAK–STAT pathway in PC.^84^ It was also reported that MUC16 might correspond with the up‐regulation of other mucins, such as MUC1 and MUC4 in PC.^85^ Overall, the involvement of MUC16 in the carcinogenesis of PC has been widely identified.

### 4.5 Others

Mucins in pancreatic disease, particularly MUC1, ‐4, ‐5 and ‐16 have gained extensive attention because of their important roles in carcinogenesis, differential diagnosis and prognosis prediction. Other mucin family members have also been studied in the field of PC. MUC3 has cysteine‐rich domains that can inhibit apoptosis and enhance the progression of PC cells,^86^ whereas MUC6 shows no effects on patient survival in PC.^34^ MUC12 and MUC11 share sequence homologies and are found in pancreatic tissues, although there is limited evidence.^5^ Few studies of MUC15, ‐17 and ‐20 have been published, although some reports have revealed that their expression in PC might exceed that in the normal pancreas.^87^, ^88^

## 5 ROLE OF MUCIN IN THE CARCINOGENESIS OF PANCREATIC CANCER

Although the oncogenic mechanism underlying pancreatic malignancy remains unclear, several studies have identified the critical role of mucins in PC formation, such as prompting tumorigenicity, enhancing metastasis and producing chemoresistance by O (or N)‐linked oligosaccharides.^37^ In summary, mucin‐mediated interactions, oncogenic signalling pathways and genetic alterations contribute to PC carcinogenesis.

### 5.1 Molecular mechanisms of mucin in pancreatic cancer

Mucins have important roles at different stages of PC by altering their expression and glycosylation during the transition from healthy tissue to neoplasia in the pancreas. Apical mucins are brought into proximity with receptor tyrosine protein kinases (RTKs) through the loss of polarity of epithelial cells. RTKs are important in signalling pathways that promote cell proliferation and cancer metastases.^46^, ^89^ The nuclear localization of mucins is associated with poorly differentiated cancers, high metastasis and unfavourable prognosis.^90^, ^91^, ^92^ Mucins also modify the exfoliation of primary cancer cells,^93^, ^94^ accelerate cellular migration,^15^ enhance proliferation^95^ and act as mediators of cellular adhesion during metastasis.^96^ The pancreatic tumour microenvironment (TME) contains fibroblasts, pancreatic stellate cells, collagen, fibronectin and some cytokines, and importantly, mucin has many interactions with the TME to mediate immune evasion, oncogenic signalling and angiogenesis.^93^, ^97^

### 5.2 Mucin‐related signalling pathways in pancreatic cancer

Transmembrane mucins participate in various signalling pathways to promote carcinogenesis and pathogenesis, either via the extracellular domain or by directly interacting with receptors.^32^, ^46^ MUC1‐C, a powerful and representative mucin because of the presence of multiple phosphorylation sites, represents a hub protein of different signalling cascades, including the c‐SRC‐mediated signalling pathway, RAS‐ERK pathway, LEF/TCF‐dependent Wnt pathway, p53 pathway, MAP kinase pathway and HIF‐1α pathway.^39^, ^46^, ^50^, ^54^ In addition, MUC4 is gaining attention as an important molecule and is reported to bind with ERBB2 to activate the PI3K‐AKT and RAS‐ERK pathways.^46^, ^63^ In addition, MUC16 was verified to enhance tumour progression and immune regulation through the mTOR, c‐MYC, MAPK and JAK‐STAT pathways.^82^, ^83^, ^84^ These roles of mucins have been comprehensively reviewed elsewhere.

### 5.3 Genetic alteration of mucins

A pan‐mucin genomic study across multiple cancers was conducted to identify novel genomic alterations in mucins and their functions in carcinogenesis based on The Cancer Genome Atlas (TGCA) dataset. DNA mutational analysis revealed the T112P mutation in 50% of stage II pancreatic *MUC1* mutations. MUC16 also has a high mutational rate in cancers, and amino acid‐altering mutations were observed in 43% of PC specimens, with 14.9% leading to deletions or frameshifts.^98^

## 6 MUCIN AS A DIAGNOSTIC TOOL IN THE DETECTION OF PANCREATIC CANCER

Most patients with PC are diagnosed at an advanced stage, especially in borderline resectable stages or metastases. Therefore, sensitive diagnostic markers for early‐stage disease are urgently required. The presence of sialylated Lewis antigen on the surface of mucins is related to the advanced stages of malignant tumours.^99^ CA19‐9 recognizes the sialylated Lewis a/b antigen on MUC1. CA19‐9 remains the only marker recommended by the Food and Drug Administration (FDA) in the USA for monitoring PC. However, nearly 10%‐20% patients are Lewis a/b‐negative, resulting in a sensitivity of less 95%.^100^ To further enhance the diagnostic value of CA19‐9, it is combined with other mucins such as MUC5AC.^64^, ^72^

Additionally, endoscopic ultrasonography‐guided, fine‐needle aspiration (EUS‐FNA) is also applied to the diagnosis before surgery as a minimally invasive method. MUC16 and MUC4 have high specificity in diagnosing PDAC from healthy controls with 63% and 67% sensitivity by EUS‐FNA, respectively.^101^ The latest research showed that MUC4 levels in the cystic fluid of IPMN patients could accurately discriminate high‐ from low‐risk cystic neoplasms with high sensitivity.^102^ In another study of EUS–FNA, up‐regulation of MUC1 (77.5%), MUC2 (10.0%) and MUC5AC (80.0%) was reported in patients with PC, and positive ratios of MUC1 (25.0%), MUC2 (31.3%) and MUC5AC (43.8%) were detected in benign pancreatic diseases, respectively.^103^ Moreover, MUC7 was detected in aspirated material from 73% PC patients, indicating its potential as a biomarker to identify PC.^104^

In addition to EUS biopsy, circulating tumour cells have gained increasing attention as potential biomarkers for diagnosis. With respect to biomarkers in circulating blood, MUC16 has been shown to be effective in predicting recurrent disease, while overexpressed MUC4 indicates early metastasis.^105^, ^106^ Even in pancreatic juice, MUC1, MUC4, MUC5AC, MUC6 and MUC16 have been identified as candidate biomarkers for PC through detection of vesicle‐associated proteins.^107^ Analysis of mucin expression as well as other biomarkers contributes to providing superior diagnostic information and enhanced monitoring of risk of disease recurrence.

## 7 MUCINS AS PROGNOSTIC INDICATORS IN PANCREATIC CANCER

**FIGURE 2:**
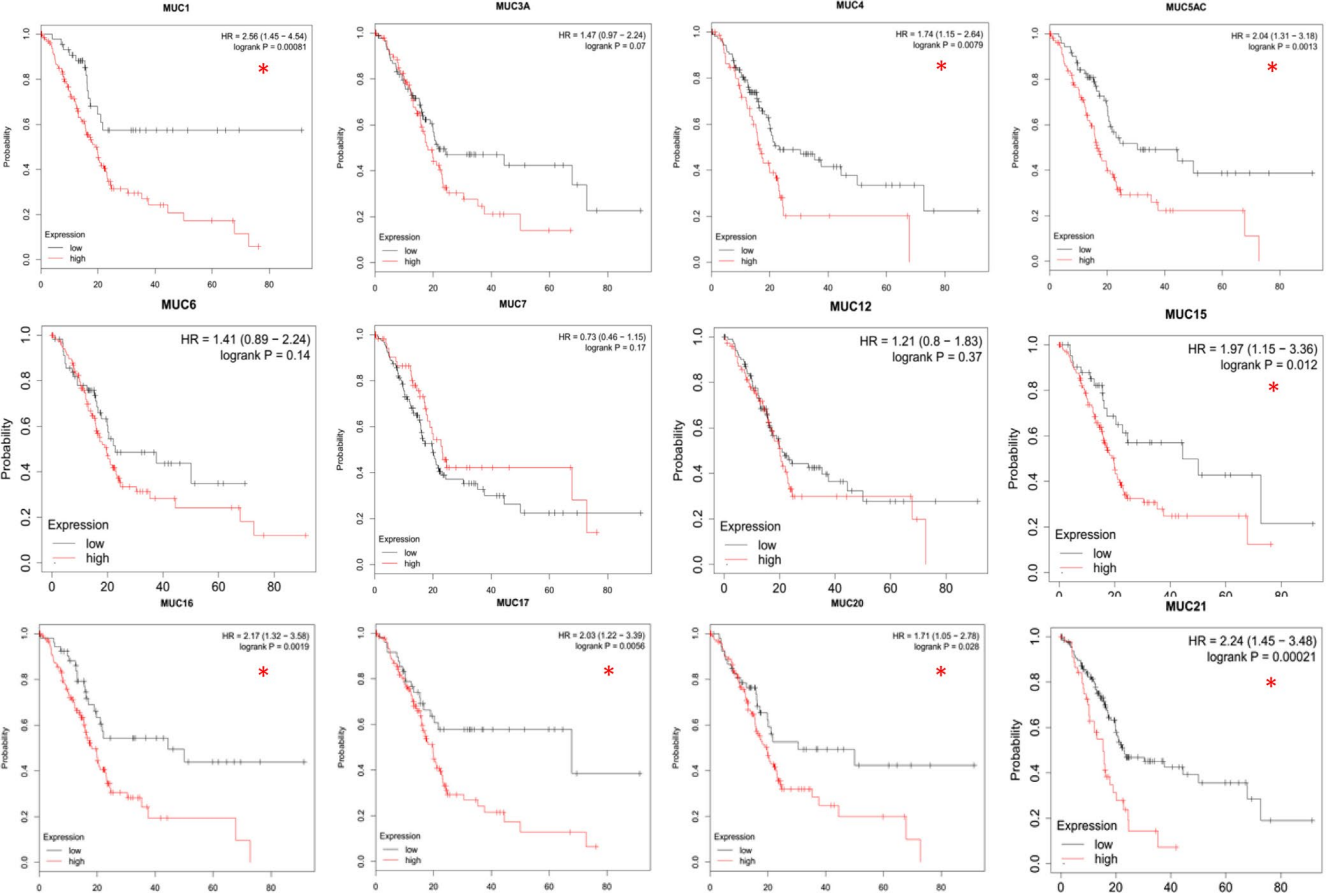
The overall survival curves of mucins in pancreatic cancer depicted by Kaplan‐Meier plotter (*P < .05)

The overall survival curves associated with members of the mucin family were depicted by online Kaplan‐Meier plotter (www.kmplot.com), a novel interactive website that aims to compare gene expression levels and prognostic conditions according to TCGA. The median was selected as the group cut‐off value for survival plots. Correlations between the mRNA levels of the mucin family and the overall survival of patients with PC is shown in Figure 2. Among the available data, 12 mucins were analysed, eight of which were significantly associated with prognosis: MUC1, MUC4, MUC5AC, MUC15, MUC 16, MUC17, MUC20 and MUC 21. However, further studies are required to consider the impact of pathological stage, sex and degree of differentiation on the prognostic value of mucin. Additionally, although the relationship between mRNA expression and prognosis has been clarified, further analyses are needed at the protein level. Considering the important roles played by mucins in PC and their prognostic value as determined using the online database, it is necessary to evaluate the function of a panel of multiple mucins for PC.

## 8 MUCIN‐BASED THERAPEUTIC STRATEGIES

**FIGURE 3:**
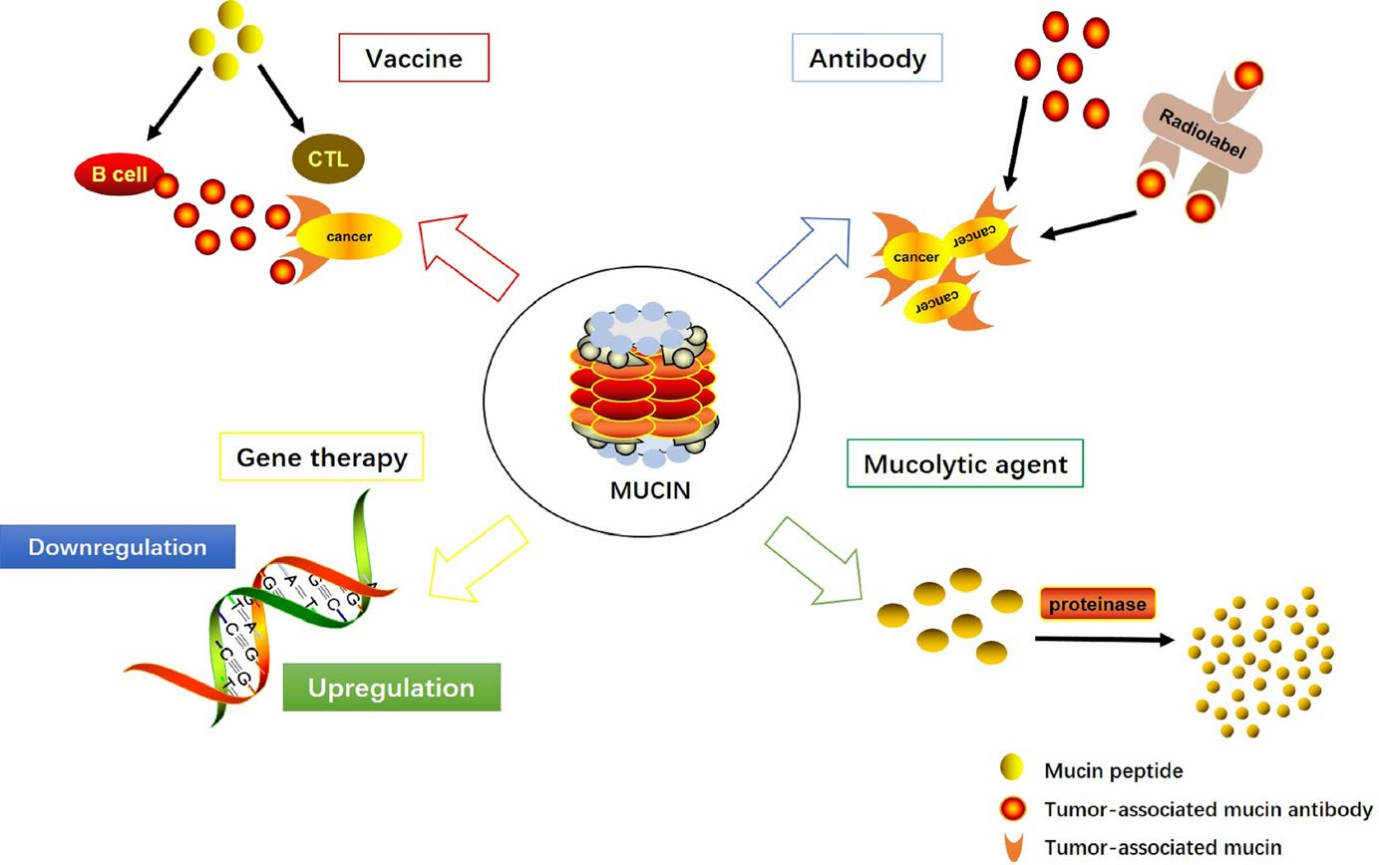
Mucins play important roles in the therapy of pancreatic cancer. The red arrow indicates the application of mucin‐based vaccines. The blue arrow shows the antibody targeting mucins for therapy. The yellow arrow implicates the gene therapy by altering the expression of mucins. The green arrow manifests the mechanism of mucolytic agents

Mucins are involved in many malignant processes including evasion, invasion and metastatic by affecting oncogenic signalling, cell survival, proliferation and resistance to chemotherapeutics.^38^, ^62^ Furthermore, several mucins have been linked with tumour progression, chemoresistance and prognosis in PC. Because of these attributes, mucin‐based therapy has also been applied for PC strategies including vaccines, antibodies, gene therapy and mucolytic agents (Figure 3).

### 8.1 Vaccines

MUC1 has garnered great interest during the course of mucin research, and the clinical application of MUC1 as a vaccine for activating the immune response against tumour‐associated antigens has been investigated. Interestingly, MUC1‐specific cytotoxic T‐lymphocytes prompted clinical studies of MUC1‐targeting vaccines in PC, and a subsequent animal experiment revealed that MUC1 could provide immune protection against cancer cells in wild‐type mice.^108^ The relationship between MUC1 and T‐cell numbers are important in the immune response.^109^ Many vaccine formulations of MUC1 have been administered to advanced‐stage patients. For example, a vaccine using MUC1‐TRR peptide,^110^ a synthetic MUC1 peptide carried by Bacillus Calmette‐Guérin^111^ or even Freund's adjuvant^112^ is used to strengthen the antigen presentation of dendritic cells and activate cytotoxic T cells. Further mucin‐specific cytotoxic T‐lymphocytes with no isotype‐transforming antibody response continue to be investigated. Many experiments of MUC1 peptide‐loaded dendritic cells were conducted in subsequent clinical trial phases, revealing that this novel vaccine preparation could be well tolerated without recurrence.^113^ Recently, MUC1‐based glycosylated tricomponent antitumour vaccines demonstrated a clear reduction in tumour burden by eliciting both cellular and humoral immune reactions.^114^ Furthermore, MUC4‐targeted cancer therapies have been reported and many prospective immunotherapies have been considered.^67^ Vaccines linked with HLA‐A1 and HLA‐A2 MUC4 epitopes activate cellular immunity and humoral immunity.^115^ In addition, the existence of MUC4 splice variants, autoantibodies against abnormally glycosylated MUC4 and T‐cell clones against MUC4 mutations strengthen its importance as a tumour‐associated antigen in vaccine research.^67^

### 8.2 Antibodies

Some anticancer therapeutics based on mucin antibody binding to tumour antigens or targeting PC have also been explored. A new MUC1 antibody named PankoMab (Berlin, Germany) is considered to have beneficial features.^116^ PankoMab can specifically recognize the carbohydrate‐induced conformational epitope of PC, differentiating between malignant and non‐malignant mucin‐related antigens while producing huge antibody‐dependent killing responses. It also stimulates MUC1‐expressing PC cells to produce strong antibody‐dependent cytotoxicity. Even for cancers originating from glandular cells or squamous epithelia, the humanized form of PankoMab showed a strong response in an IHC analysis of 137 surgical specimens.^117^ Another way to eliminate cancer cells is the combination of MUC1 antibodies and radioisotopes. Radiolabeled antibody PAM4, which specifically targets MUC1, has been studied in PC.^118^ Tumour targeting by ^131^iodine (131I)‐PAM4, 99mTechnetium (99mTc)‐PAM4, and the humanized form of 90Y‐PAM4 (h90Y‐PAM4) was shown to be partially effective with acceptable adverse effects.^119^, ^120^ And an α‐immunoconjugate of a monoclonal antibody, also known as C595, which can discern the TRR on MUC1, has been adopted as a valuable approach.^121^ However, although some evidence for their effectiveness is promising, the side effects of radiotherapy such as neutropenia and thrombocytopenia as well as the high cost should be considered.^122^

### 8.3 Gene therapy

Several ongoing clinical studies proved that down‐regulation of the expression of selected mucins could be a new therapeutic approach for PC. Knockdown of *MUC1* by short interfering RNA inhibited the proliferation of PC cell lines, and injection of these cells into the pancreatic tissue of an animal model reduced the incidence of metastasis.^123^ Similarly, *MUC4* interference by siRNA in CD18/HPAF PC cells also inhibited proliferation in an orthotopic mouse model.^90^ Except for targeting down‐regulated mucins, the mucin gene promoter also will induce tumour cell death in MUC1‐expressing PC.^124^ Although some studies showed the potential effect of gene therapies, they neglected to address the challenge of delivering the gene into the human body and are also not clinically approved.

### 8.4 Mucolytic agents

It has been reported that changes in the dense mucin mesh could alter the absorption of cytotoxic drugs. Mucins constitute a barrier on the cell surface that can infect drug uptake.^125^ Therefore, drug‐dissolving mucins such as bromelain and N‐acetylcysteine (NAC) have been applied.^126^, ^127^ Bromelain is a cysteine proteinase composed of thiol endopeptidases that destroy the glycosidic bonds of mucins, while NAC denatures the disulphide bonds of mucus to decrease mucus viscosity, facilitating its clearance.^128^ The combination of such two drugs can be used as an adjunct to cytotoxic drugs because of its effect on the lysis of mucin on the cell surface.^129^

## 9 CONCLUSIONS

Pancreatic adenocarcinoma is a worldwide challenge with high rates of incidence and mortality. Although many efforts have been made to identify the main mechanism of PC occurrence and development, the exact molecular processes underlying the aggressive nature of this devastating disease remain to be elucidated. Mucins, well‐known and promising glycoproteins, have been investigated in the pathogenesis and progression of this malignancy, and their expression is useful in the differential diagnosis of PC, premalignant lesions and benign pancreatic tumours. Mucins have been identified as being more valuable in ameliorating the unfavourable survival status of PC than previously thought. Moreover, they also participate in pancreatic carcinogenesis, indicating their significance as future therapeutic targets.

Because the association between PC and mucins has been studied for several years both in the laboratory and in clinical trials, it is clear that the mucin family has significant functions in the carcinogenesis of PC and in therapy targeting PC. In addition, the potential functions of other mucins contributing to the carcinogenesis of PC, such as MUC4, MUC5 and MUC16, have also recently been investigated in detail. Although the roles of MUC1, MUC4, MUC5AC and MUC16 in PC have already been described, evidence for the significance of other mucins is still not available, and new members of the mucin family are likely to be identified. However, no mucin alone can enhance the entire biological process of PC, and optimal therapy for PC could consist of combined agents that target a series of mucins. In conclusion, the data presented in this review provide insights into the utilization of mucin family members as biomarkers and therapeutic targets in PC, and indicate further potential research areas examining new functional roles, biomarker functions and therapeutic drugs against mucins in PC.

## CONFLICT OF INTEREST

The authors confirm that there are no conflicts of interest.

## AUTHOR CONTRIBUTIONS

**Shunda Wang:** Data curation (lead); Formal analysis (lead); Methodology (lead); Writing‐original draft (lead). **Lei You:** Visualization (lead). **Menghua Dai:** Conceptualization (equal); Supervision (equal). **Yupei Zhao:** Conceptualization (equal); Supervision (equal).
